# Damage Analysis of Composite CFRP Tubes Using Acoustic Emission Monitoring and Pattern Recognition Approach

**DOI:** 10.3390/ma14040786

**Published:** 2021-02-07

**Authors:** Michal Šofer, Jakub Cienciala, Martin Fusek, Pavel Pavlíček, Richard Moravec

**Affiliations:** 1 Department of Applied Mechanics, Faculty of Mechanical Engineering, VŠB—Technical University of Ostrava, 17. listopadu 2172/15, 708 00 Ostrava, Czech Republic; jakub.cienciala@vsb.cz (J.C.); martin.fusek@vsb.cz (M.F.); pavel.pavlicek@vsb.cz (P.P.); 2 Havel Composites CZ s.r.o., Svésedlice 67, 783 54 Přáslavice, Czech Republic; moravecr@havel-composites.com

**Keywords:** acoustic emission, CFRP composite tube, unsupervised learning approach, failure mechanism

## Abstract

The acoustic emission method has been adopted for detection of damage mechanisms in carbon-fiber-reinforced polymer composite tubes during the three-point bending test. The damage evolution process of the individual samples has been monitored using the acoustic emission method, which is one of the non-destructive methods. The obtained data were then subjected to a two-step technique, which combines the unsupervised pattern recognition approach utilizing the short-time frequency spectra with the boundary curve enabling the already clustered data to be additionally filtered. The boundary curve identification has been carried out on the basis of preliminary tensile tests of the carbon fiber sheafs, where, by overlapping the force versus time dependency by the acoustic emission activity versus time dependency, it was possible to identify the boundary which will separate the signals originating from the fiber break from unwanted secondary sources. The application of the presented two-step method resulted in the identification of the failure mechanisms such as matrix cracking, fiber break, decohesion, and debonding. Besides the comparison of the results with already published research papers, the study presents the comprehensive parametric acoustic emission signal analysis of the individual clusters.

## 1. Introduction

Over the past decades, carbon-fiber-reinforced polymer (CFRP) composites have shown a constant increase in a variety of applications such as car or aircraft components, sports and medical equipment [1], and recently also additive manufacturing [2,3,4]. Their main benefit lies primarily in the relatively high strength/weight ratio or the ability to customize the material properties for dedicated purposes by changing the stacking sequence and related fiber orientation. A relative drawback of CRFP composites is the lack of ductile-like behavior and the corresponding absence of pre-warning phase before the structural collapse [5,6] leading to the brittle failure. CRFP composites are also characterized by the accumulation of damage inside the structure without any evidence on the structure surface [6] thus leading to a relatively challenging damage assessment. There are many non-destructive testing approaches, which can be applied on composite structures, namely infrared tomography [7], eddy current testing [8], ultrasonic testing [9], and X-ray tomography [10]. 

One of the most promising approaches, especially coupled with other methods [11] such as Scanning Electron Microscopy (SEM), is the acoustic emission (AE) method, which is also used in various applications as a real time monitoring tool [6]. The AE method exhibits great sensitivity including considerable reliability of active cracks detection [12], even in the case of initiation phase [13]. The AE technique is even capable of detecting the onset of plastic deformation [14], which has the character of white noise with low energy [15]. For gaining a more detailed insight into the damage monitoring process within the meaning of AE source characterization, it is favorable to incorporate an adequate signal analysis tool. The supervised/unsupervised pattern recognition (UPR) approach [16] has become a very suitable and promising approach to tackle a wide variety of problems such as fatigue tests [17], structure health monitoring [18], and condition assessment of pressure vessels [19] and pressure components in operation [20]. Numerous studies [21,22,23,24,25,26] have been conducted in order to assess characteristic features of the AE transients originating from various failure mechanism in the CFRP composites such as matrix cracking, delamination, fiber break, and debonding (see Table 1 for further explanation). 

Although Chou [21] points to a discrepancy concerning, in particular, the signal amplitude, duration as well as frequency spectra of the individual damage mechanisms, it was possible to compile a general overview, which is given in the following table (Table 2).

In the last decade, several/numerous studies utilizing advanced techniques for classification of failure modes, such as the use of statistical analysis of wavelet coefficients [27] or infrared thermography (IT) [28], have been conducted. Another interesting approach can be found in the work published by Munoz et al. [29], who identified and further characterized the damage mechanisms in the unidirectional CFRP composites subjected to axis and off-axis static tensile tests using the acoustic emission method and infrared thermography. Further utilization of unsupervised pattern recognition technique together with the IT method resulted in the identification of the failure mechanisms such as matrix cracking, fiber breakage, and interface failure, for which the characterization in terms of the signal amplitude or energy has been performed. In 2011, Gutkin et al. published an extensive research [30], in which the AE signal data from various test configurations were analyzed by three different pattern recognition approaches. The analysis resulted in characteristic frequency spectra for matrix cracking, delamination, debonding, fiber pull-out, and fiber failure. It has to be noted that the given findings in terms of the frequency spectra are to some extent similar to the results summarized in Table 2 and therefore confirms the factual accuracy of the study [30]. 

The main objective of this study is to investigate and comprehensively describe the AE signal characteristics of the damage mechanisms in three different types of CFRP composite tubes using a two-step method combining the unsupervised pattern recognition approach with the utilization of the boundary curve. The construction of the boundary curve has been conducted on the data from the preliminary carbon fiber sheaf tensile tests. The already identified boundary curve has then been used for further refinement of the data across individual clusters. Using the presented approach, it was possible to identify a total of four damage mechanisms presented in Table 1 with subsequent comparison of the obtained results with the already published research papers. The part of the study is also the comprehensive AE waveform analysis of the representative signals belonging to the individual clusters. 

## 2. Experimental Procedure 

### 2.1. Test Sample Characterization

**Figure 1:**
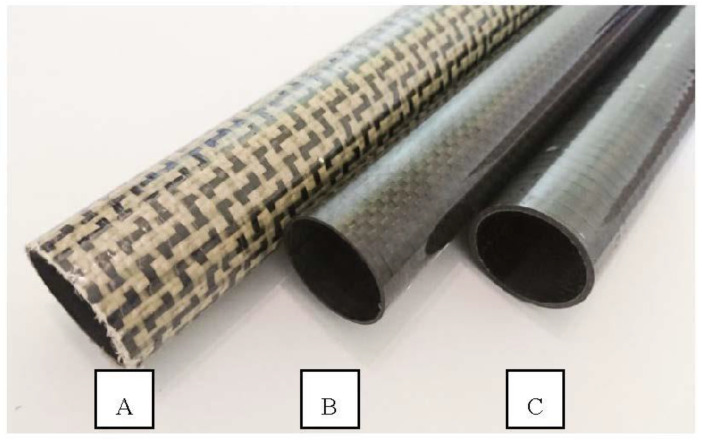
CFRP composite tubes under test; A—A series sample, B—B series sample, C—C series sample.

The experiments were carried out on three types of CFRP tubes with a different number of layers, their orientation, and woven fiber density of the used material (see Figure 1), where each type of CFRP tube has been represented by three test samples. The samples labeled “A” were manufactured using four layers of unidirectional carbon woven fabric with density of 200 g/m^2^ and one layer of aramid/carbon woven fabric (0–90°) with density of 175 g/m^2^ and average wall thickness of 1.45 mm. The production of samples labeled “B” included the use of two layers of unidirectional carbon woven fabric with density 300 g/m^2^ and one layer of carbon woven fabric (0–90°) with density 280 g/m^2^ with average wall thickness of 0.9 mm, while samples labeled “C” were manufactured using solely four layers of unidirectional carbon woven fabric with density of 300 g/m^2^ with average wall thickness of 1.42 mm. Table 3 summarizes the specification of the tested CFRP tubes. 

### 2.2. Three-Point Bending Test

**Figure 2:**
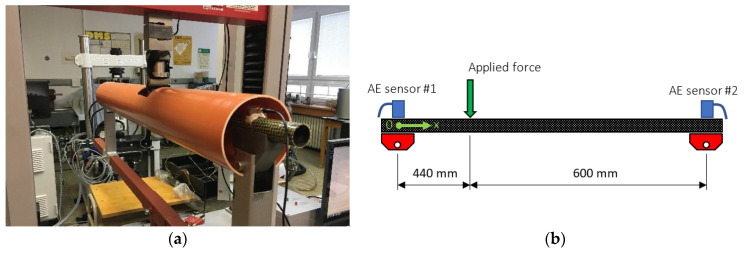
(**a**) In-Situ photograph of the test rig including the specimen equipped with AE sensors; (**b**) Schematic representation of the three-point bending test setup.

The three considered types of CFRP composite tubes under test are being used for paddle production; therefore, the three-point bending test has been selected in order to simulate as much as possible the real nature of the loading process during the use of the given sports equipment. The experiments were carried out on the universal Testometric M500-50CT testing machine (The Testometric Company Ltd., Rochdale, UK) with dedicated weldment, which enables its geometry to be modified with its moving parts for a wide variety of such experiments. The distance between the supports was equal to 1040 mm with the force acting point in a distance of 440 mm from AE sensor #1 (see Figure 2). The tested CFRP tube with attached AE sensors was additionally placed in a plastic pipe to prevent damage to the AE sensors and other equipment due to sudden structural integrity violation. The supports were covered by thin felt to allow free movement of the tube during its bending. The test has been deformation-controlled with the upper anvil speed equal to 10 mm/min.

### 2.3. Acoustic Emission Monitoring

The acoustic emission activity has been monitored using the Vallen AMSY-6 AE system (Vallen Systeme GmbH, Icking, Germany) with two utilized measuring channels (ASIP-2A dual channel signal processor card), equipped with the AEP5H 34 dB preamplifiers and the broad-band Vallen VS-900 AE sensors. The sensors were attached onto the tube with the use of oil-based plasticine. The sampling frequency of the AE data was set to 10 MHz while the transient data (wave transients) were sampled with 20 MHz in the frequency range between 50 and 1100 kHz. The detection threshold has been set to 32 dB owing to a relatively greater distance of both sensors from the area in which the breach will most likely occur and related higher attenuation of the AE signal in composites. Only localized AE events, which fall within the 〈250,650〉 (mm) (see Figure 2b) interval of the x coordinate will be included for further data processing.

**Figure 3:**
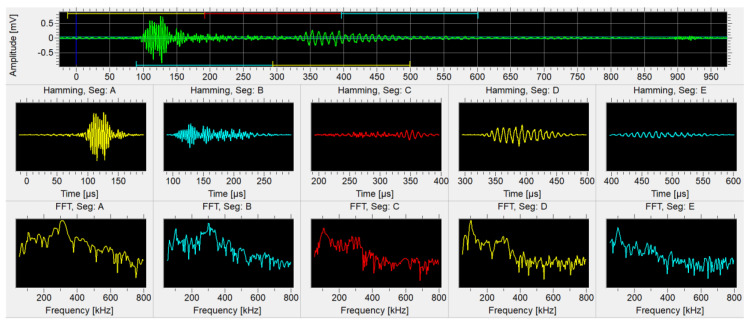
Setting up the Hamming windowed time segments with corresponding results in the frequency domain.

The filtered data was followingly analyzed with the Vallen VisualClass software package (Vallen Systeme GmbH, Icking, Germany), which uses the pattern recognition method [15] to associate similar waveform types into separate groups. Due to the nature of the task, an unsupervised learning approach was chosen. The procedure starts with loading the selected database of AE transients into Vallen VisualClass software, where the number of time windows including their span and the starting point of the segmentation analysis in the time domain must be specified (see Figure 3).

**Figure 4:**
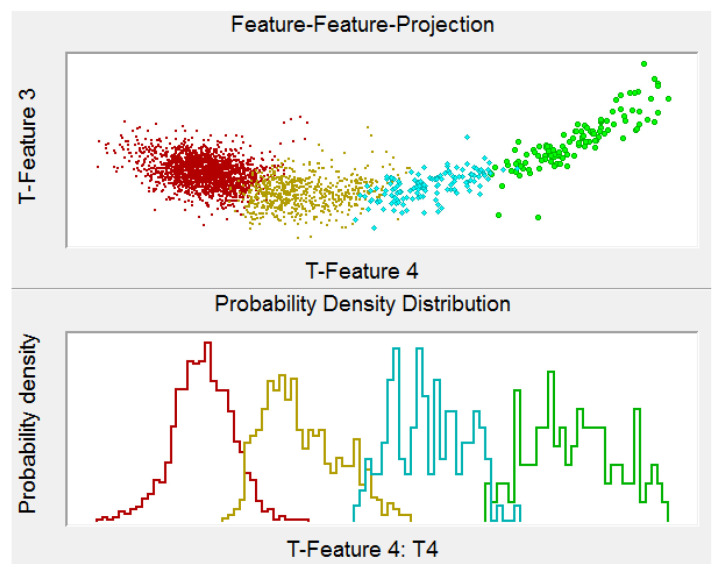
Transformed features projection—results for four selected clusters.

The current analysis uses the following settings for the AE transients with relation to the VisualClass software package: Number of time segments: 5; Size of single time segment in terms of points: 4096; Rel. trigger offset: −256 points; min/max frequency limit: 50/800 kHz. The software then performs the assembly of multidimensional feature vector, the size of which depends on the chosen number of time segments including their size. The pattern recognition analysis will result in the basic feature space identification, which is then linearly transformed for maximizing inter-class distance and minimizing the intra-class extension, at the same time (see Figure 4).

The results are then transferred into VisualAE software for further postprocessing. Four clusters were chosen for subsequent analysis, since the additional increase of the number of clusters did not lead to better differentiation of individual transients. One sample from each series has been subjected to the attenuation measurement of the AE signal using Hsu-Nielsen source [31] (pencil lead diameter: 0.35 mm; hardness: 2H) in order to properly evaluate the real AE signal amplitude in subsequent data analysis. The results of the attenuation measurements are shown in Table 4. The propagation velocity has been determined experimentally using Hsu-Nielsen source with value varying between 3200 and 3300 m/s across A/B/C series samples.

**Figure 5:**
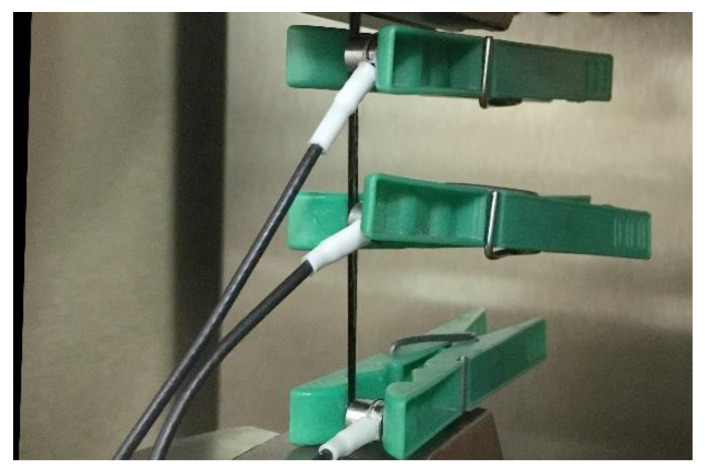
Experimental setup for tensile test of the carbon fibers.

The preliminary measurements also included a series of six tensile tests of carbon fiber sheafs (see Figure 5) in order to construct the above-mentioned boundary curve, which will be further used for the detection of the carbon fiber breaks across the identified clusters. The deformation-controlled tests (upper anvil speed equal to 0.5 mm/min) were carried out on the universal Testometric M500-50CT testing machine equipped with 100 N load cell. The relatively small scale of the load cell enabled us to detect in time the events corresponding to the failure of certain number of fibers thanks to the registered force drop. The AE activity has been monitored using the Vallen AMSY-6 AE system with three utilized measuring channels (ASIP-2S dual channel signal processor card), equipped with the AEP5H 34 dB preamplifiers and the broad-band DAKEL MIDI AE sensors, where the top and bottom sensor acted as guard elements, while the middle sensor has been used for the data acquisition. The fiber sheafs were glued on their ends thus providing a clamping support for the attachment into the jaws.

The basic frequency analysis of the AE signal is, besides the exploited pattern recognition approach, relatively efficient and powerful tool for filtering the AE signal, which can be then affiliated to different failure mechanisms [32]. Chou in his work [21] states that fiber breakages in the case of carbon fiber/glass fiber composite systems produce extensional wave signals with frequencies between 350–700 kHz, while matrix cracks generate flexural wave modes with frequencies up to 350 kHz. It has to be noted that the given finding has been verified on the preliminary tensile tests of the carbon fibers, where the AE signals originating from fiber failure exhibit a higher power fraction in the 300(350)–600 kHz frequency interval. Based on this consideration, there will be defined a variable denoted as p_f_ factor, which will relate the power fraction of the AE signal in a certain frequency band to the AE signal power in the entire considered frequency range:

(1) $$P_{f}=\frac{P_{(350–800)\mathrm{kHz}}}{P_{(50–800)\mathrm{kHz}}}100\left(\%\right)$$

where P_(350–800)kHz_ represents the power fraction of the AE signal in the 350–800 kHz frequency range and P_(50–800)kHz_ is the power of the AE signal in the entire considered frequency range, i.e., 50–800 kHz. Note that the power of the AE signal in the given frequency interval has been calculated using Parseval’s theorem. The boundary curve then states the p_f_ factor and the AE signal amplitude in relation with subsequent intention to filter the AE signal originating from the fiber failure from the other failure mechanisms or the secondary AE signals, which figure as noise (interaction of the individual fibers between each other (rubbing) and/or AE activity arising from the sample attachment points). The identification procedure of the boundary curve is based on the overlapping the force versus time dependency by the acoustic emission activity versus time dependency, where the detected force drops caused by the failure of the individual/multiple fibers can be directly matched to the emerged AE signals.

**Figure 6:**
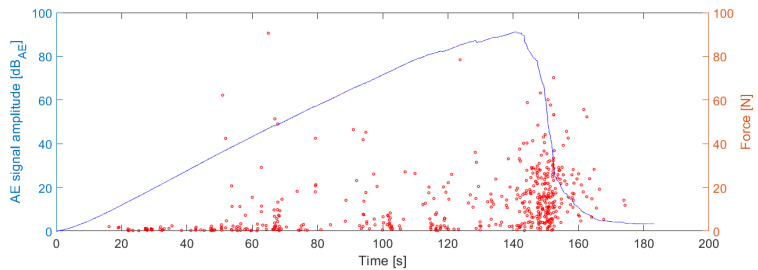
Force and AE activity as the function of time for the selected sample.

**Figure 7:**
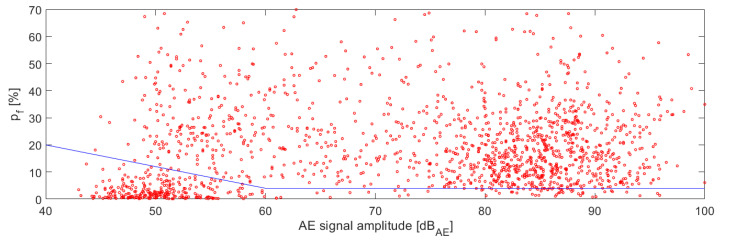
Dependency between p_f_ factor and AE signal amplitude for the entire series.

The following figures display the dependency between the force and amplitude of individual AE hits on time (Figure 6) and the relation between the p_f_ factor and the AE signal amplitude for the entire series (Figure 7). Note that the AE signal amplitude is being referred to dB_AE_ unit, thus expressing the voltage amplitude of non-amplified signal as a gain related to 1 µV. The blue line in Figure 7 represents the boundary line separating AE signals belonging to the fiber break from signals originating from the interaction between the individual fibers and the other interfering AE sources. At this point, it has to be noted that the high-frequency range appearing in numerator in Equation (1) has been for further application of the CFRP tubes extended to 300–800 kHz range due to a frequency attenuation arising from the geometric dimensions of the CFRP tubes and the mutual distance between the AE source and the AE sensors.

## 3. Results and Discussion

### 3.1. Mechanical Properties and Basic AE Signal Analysis

**Figure 8:**
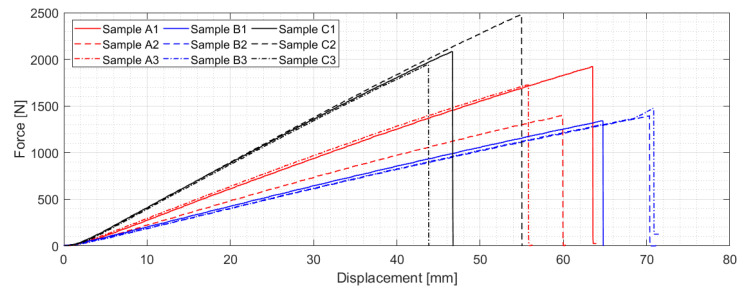
Force as the function of displacement for individual A/B/C series production samples

**Figure 9:**
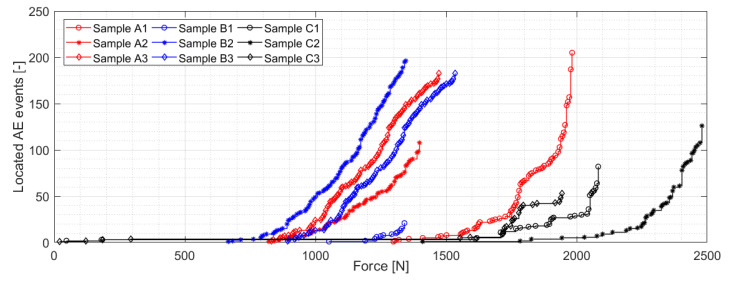
Cumulative number of located events as the function of force for individual A/B/C series samples

Figure 8 shows relation between force and displacement of the anvil for individual A/B/C series production samples. As expected, the highest stiffness is being reached by the C series samples, followed by A and B series samples. All three manufacturing modifications show within their group very similar trend in terms of the force-displacement course except the A series samples, namely A2 sample, which exhibits marginally lower stiffness, most likely due to the fabrication process, which is not in the form of the automated production. The above-mentioned consistency in terms of the sample stiffness is, however, not valid for the maximum force across individual series, where differences from 10 to 28 percent related to the maximum achieved force in each production series can be observed. Again, the reason for such results variation can be found in the production form itself. A somewhat similar trend can be registered in the case of the number of located AE events across the 0–F_max_ range for the individual samples (see Figure 9), where a relatively large variation has been registered. However, even despite this fact, A/B series report considerably higher level of the located AE events, which is most likely caused due to the presence of (0°–90°) fabric figuring as a top layer. Note that the given statement is currently a hypothesis, which needs to be verified in the future.

**Figure 10:**
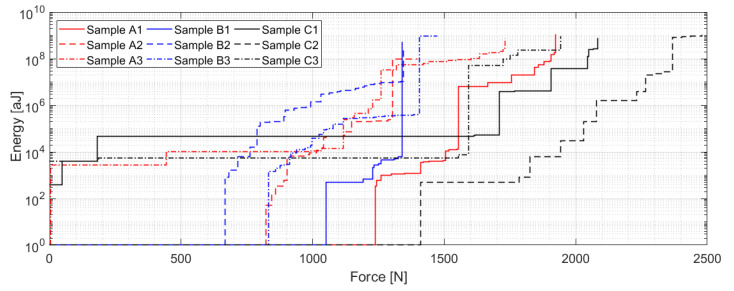
Energy as the function of force for individual A/B/C series samples

The energy of accumulated AE events versus force (Figure 10) is another important dependency, which can bring us closer to the overall structure behavior. The maximum value of the released AE energy is for all samples between 5 × 10^8^ and 10^9^ aJ. The difference, however, lies in the character how the energy is being released during the loading process. The A and B series specimen exhibit almost gradual AE energy release, with the difference in the final loading stage. While the A series specimen tend to gradually continue with the cumulation of the AE events and gradual release of the AE energy, the B series samples tend to suddenly lose integrity without any significant warning phase. A completely different behavior can be found in the case of the C series samples, which have considerably larger energy per event ratio with a lack of any warning phase before the integrity lose.

### 3.2. AE Signal Analysis Using Pattern Recognition Approach

**Figure 11:**
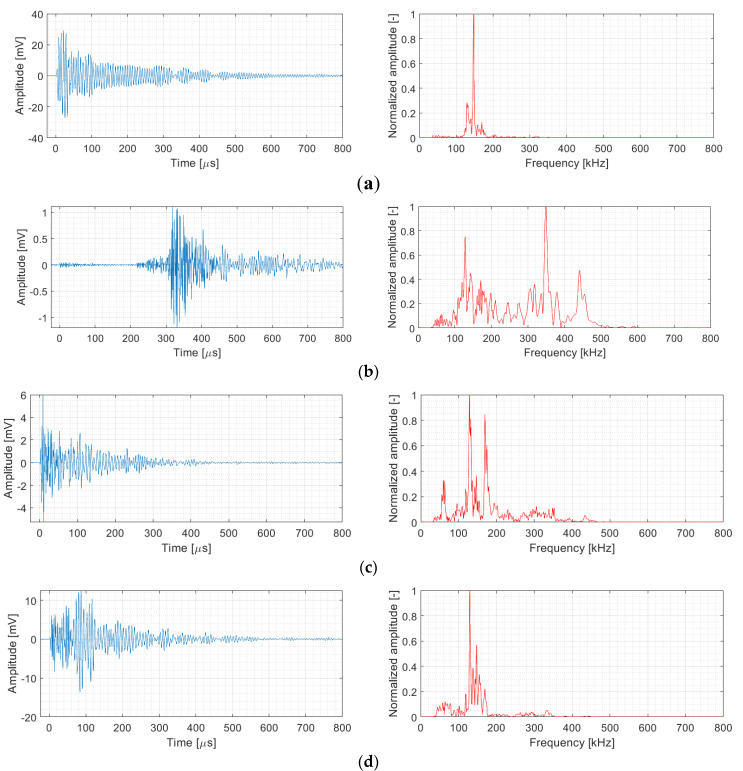
Examples of characteristic signal belonging to individual clusters: (**a**) Cluster 1; (**b**) Cluster 2; (**c**) Cluster 3; (**d**) Cluster 4.

**Figure 12:**
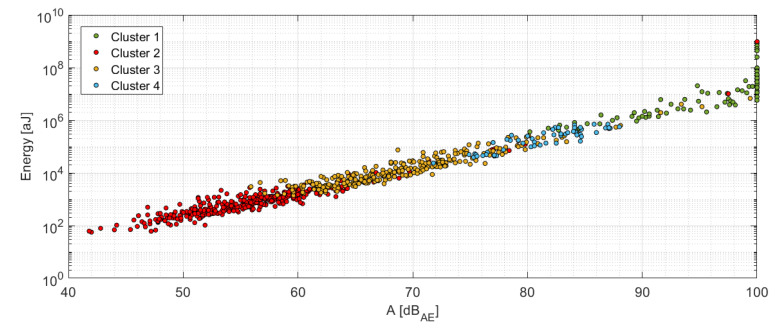
Amplitude versus Energy for individual clusters.

The utilized unsupervised pattern recognition analysis resulted in identification of four clusters of AE signals with the following features. The signals affiliated to the first cluster are characterized by high amplitude, in most cases exceeding 90 dB_AE_ with energy value usually above 1 × 10^6^ aJ and frequencies in the span from 50 kHz to 150 kHz (see Figure 11a), whereas this cluster also partially contains signals with frequency content above 300 kHz. The second cluster is characterized by the amplitudes mostly below 65 dB_AE_ with AE energy in the order of hundreds to the tens of thousands of aJ and the frequency in the 50–450 kHz range (see Figure 11b). The third cluster is represented by the signals with the amplitude in the 60–80 dB_AE_ range with AE energy in the order of ten thousand aJ and the frequency in the 50–300 kHz interval (see Figure 11c). The fourth cluster is characterized by the signals in the 90–110 dB_AE_ amplitude range, the AE energy of 1 × 10^5^–1 × 10^6^ aJ and the frequency in the 50–200 kHz range (see Figure 11d). For better clarity, the results are summarized in the following table and Figure 12, respectively.

Due to the fact that there was no 100% separation of individual clusters, it was necessary to subject the obtained cluster data to additional analysis, in which the p_f_ factor for each clustered AE event has been calculated. The subsequent separation of the events affiliated to the fiber break has been performed using the already introduced boundary curve. However, care has to be taken whether the boundary curve relates to the AE hits of the already localized AE events, where in the latter case there must be considered an additional amplitude shift, which depends on the attenuation curve for the individual sample series.

**Figure 13:**
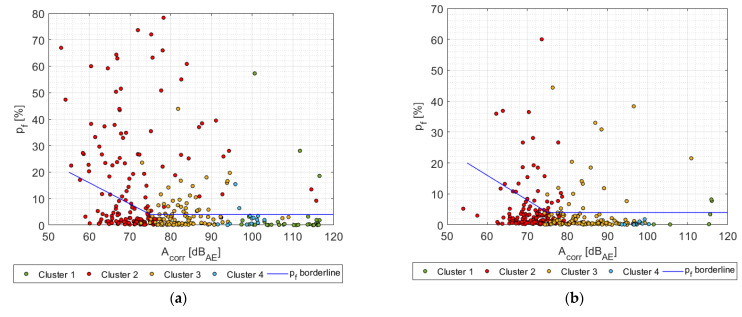

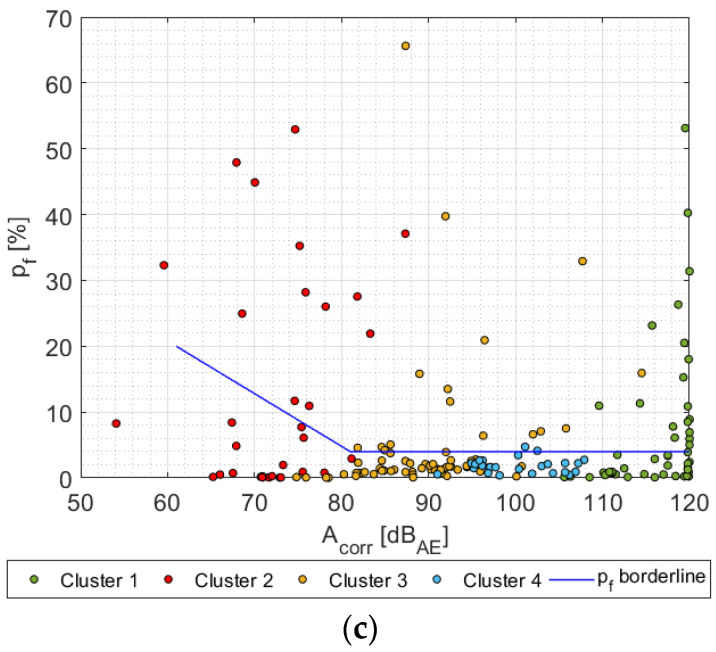
p_f_ factor as the function of distance corrected amplitude A_corr_—A series samples (**a**); B series samples (**b**); C series samples (**c**).

**Figure 14:**
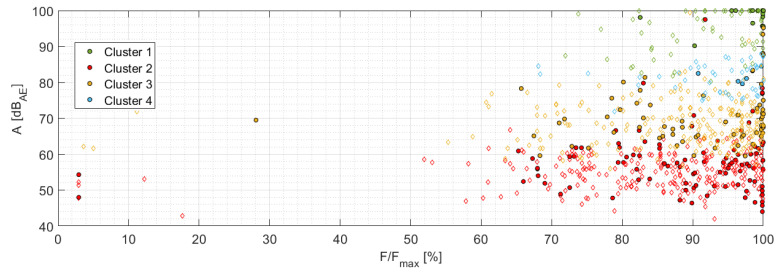
Amplitude as the function of F/F_max_—A,B,C series.

**Figure 15:**
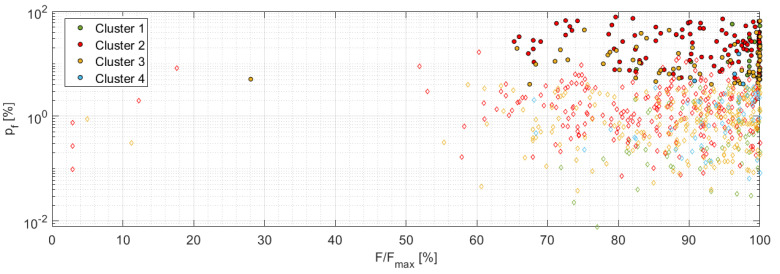
p_f_ factor as the function of F/F_max_—A,B,C series.

Figure 13 displays the resulting p_f_ = f(A_corr_,cluster) dependency for individual sample series including the drawn boundary curve, which has already been shifted using the identified attenuation curve for the given sample series and the reference distance (440 mm). Figure 14 shows the A = f(F/F_max_) dependency for the localized AE events, which were detected for all three-sample series. The filled symbols represent the fiber break while empty ones represent failure mechanism affiliated to the given cluster. For the straightforward comparison the original vertical axis, showing the distance corrected amplitude A_corr_, has been replaced by the AE signal amplitude—A in dB_AE_. Figure 15 displays the relation between the p_f_ factor and the F/F_max_ quantity for all considered series.

**Figure 16:**
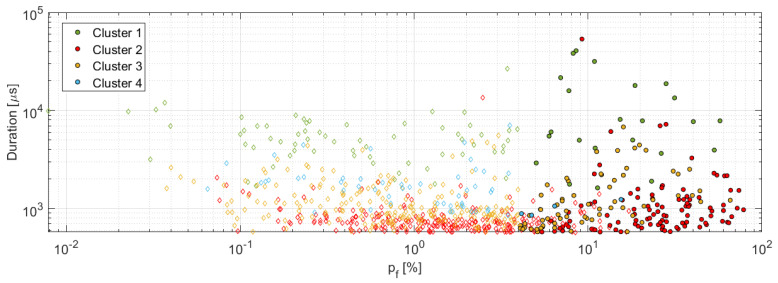
p_f_ factor versus duration—A,B,C series.

It is obvious that the damage is being initiated with the AE signals assigned to the cluster 2 with low values of the p_f_ factor at the same time at the level of 50% of the maximum force, while the first indication of the fiber break appears at 65% of the maximum force. The AE signals affiliated to the cluster 2 can be therefore assigned to the matrix cracking in the initiation phase, including the high-frequency AE signals evaluated as the fiber breakage (29.5% incidence). Classification of the cluster 2, at least its low-frequency content group, is fully in accordance with literature [25], where the amplitudes corresponding to this failure mechanism were below 70 dB. The same applies to the frequency content (see Table 5), especially its low-frequency part, which is identical to results published in [24] or [32]. The formation of the two separate groups, i.e., low- and high-frequency content, within the cluster 2 becomes more evident if the p_f_ = f(duration) dependency is displayed, see Figure 16, where the same applies also to the other clusters. Gutkin et al. [30], on the other hand, reports a lower frequency band compared to the above-mentioned research papers.

With a gradually increasing value of the F/F_max_ variable, we can register the emergence of the AE signal classified to the cluster 3 and the cluster 4, which contain 22.8% and 7.4% of the AE hits classified as the fiber break, respectively. Cluster 3 is likely to represent the debonding, while the cluster 4 affiliates to the delamination. Both these classifications are in accordance with [32] including the duration of the AE signal, which does not exceed 10 ms.

**Figure 17:**
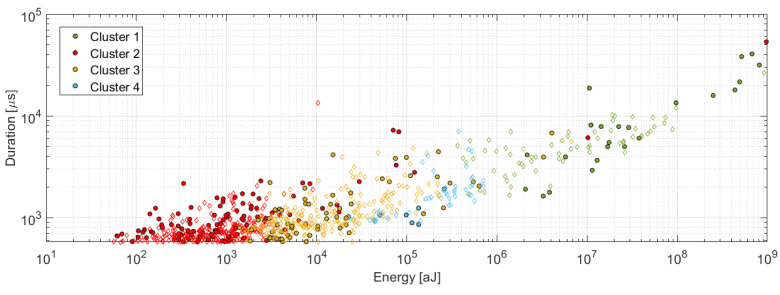
Energy versus duration—A,B,C series.

While the already obtained results indicate relatively similar frequency bands of the delamination and the debonding failure mechanism, where the delamination exhibits higher limit equal to 300 kHz (see Table 5), the results provided in [30] report a considerably lower frequency band for the delamination, which is contrary to [32] or [33]. Cluster 1, on the other hand, reflects cluster 2 to a certain extent, while having much higher levels of the AE signal energy including the amplitude. Its low- and high-frequency sections are well separated as can be seen in Figure 16. The F/F_max_ values of the AE signal belonging to this cluster are higher than 80%, thus pointing to the emergence of dominant events with loss of the structural integrity, which are represented even by propagation of the interlaminar matrix cracks or the failure of larger amounts of the carbon fibers [34]. A somewhat specific is the fiber break failure mechanism, which occurs across all clusters as a high-frequency content AE signal with amplitudes ranging from 45 to 100 dB including wide AE energy span (10^2^–10^9^ aJ), see Figure 17, and high-frequency content above 350 kHz. This finding has been supported by various research papers [22,23,30,34].

## 4. Concluding Remarks

The AE monitoring technique together with an unsupervised pattern recognition technique and the introduced boundary curve have been used to analyze the various damage mechanisms in three types of the CFRP composite tubes, which were subjected to the three-point bending test. The utilized two-step approach involved the preliminary tensile tests of the carbon fiber sheafs, which enabled the boundary curve to be designed for further identification of the AE signal originating from the fiber break. The boundary curve was then adopted on the clustered data, which has been obtained using the unsupervised pattern recognition approach, thus enabling the AE events originating from the fiber break to be additionally filtered across all the identified clusters. The conclusions obtained within the framework of this research study are summarized below.

1. Four damage mechanisms have been identified using the above-mentioned techniques, namely the fiber break, delamination, debonding, and matrix cracking. The application of the boundary curve appeared to be an effective tool for the further refinement of the results across the individual clusters.
2. The fiber break failure mechanism has been identified across all clusters resulting in the wide amplitude as well as energy span. This finding has been supported by various research projects/studies/papers.
3. It was found that the matrix cracking failure mechanism generates AE signals with the frequency band between 50 and 200 kHz. The result is in accordance with most studies; however, even in this matter a certain contradiction can be found [30].
4. The distinction between delamination/debonding failure mode seems to be a relatively challenging, since both failure mechanisms report very similar frequency spectra [21]. However, according to the presented study, we can find the difference in the energy as well as amplitude values of both mechanisms.

## Figures and Tables

**Table 1:** Basic characterization of damage mechanisms occurring in carbon-fiber-reinforced polymer (CFRP) composites.

Damage Mechanism	Characterization
Fiber break	Disintegration of single and/or multiple carbon fibers
Delamination	Separation of two adjacent plies (Interface failure)
Debonding	Integrity failure between fiber and matrix (Interface failure)
Matrix cracking	Nucleation and further propagating of (micro)cracks in the matrix

**Table 2:** Summary of the acoustic emission (AE) signal characteristics for given damage mechanisms in CFRP composites

Damage Mechanism	AE Signal Characteristics *
Fiber break	A: 50–100 dB_AE_, D: 100–10,000 µs, f = 300–700 kHz
Matrix micro cracks	A: 30–40 dB_AE_, D: <1000 µs, f = 100–250 kHz
Matrix micro cracks (propagation)	A: 40–80 dB_AE_, D: 1000–10,000 µs, f = 100–250 kHz
Delamination	A: >70 dB_AE_, D: 1000–10,000 µs, f = 250–300 kHz
Debonding	A: <60 dB_AE_, f ≅ 300 kHz

* A—amplitude, D—duration.

**Table 3:** Specification of the tested CFRP tubes

Property	A Series	B Series	C Series
Wall th. (mm)/Diameter (mm)	1.45/32	0.9/32	1.42/32
Fabrication	4 layers of 200 g/m^2^ unidir. carbon fabric1 layer of 175 g/m^2^ aramid/carbon fabric (0°–90°)	2 layers of 300 g/m^2^ unidir. carbon fabric1 layer of 280 g/m^2^ carbon fabric (0°–90°)	4 layers of 300 g/m^2^ unidir. carbon fabric

**Table 4:** Attenuation measurement on A/B/C series of CFRP composite tubes

Sample Series	Near Field Attenuation (dB/m)	Far field Attenuation (dB/m)
A	90	33.3
B	66.6	33.2
C	222.2	36.2

**Table 5:** Characterization of the individual clusters.

Cluster No.	Frequency Range (kHz)	Amplitude Range (dB_AE_)	Energy Range (aJ)
1	50–150 (>300, minor cases)	>90	>10^6^
2	50–450	<65	10^2^–10^4^
3	50–300	60–80	10^3^–10^5^
4	50–200	75–90	10^4^–10^6^

## Data Availability

Data can be provided upon request from the correspondent author.
